# Three-Year Visual, Tomographic, and Corvis ST-Derived Biomechanical Outcomes After Combined Intrastromal Ring Implantation and Corneal Collagen Cross-Linking for Keratoconus

**DOI:** 10.3390/vision10030042

**Published:** 2026-07-06

**Authors:** Radu-Nicolae Pop, Patricia Ariadna Nicula, Cristina Ariadna Nicula, Dorin Vasile Nicula, Bianca Pop

**Affiliations:** 1 OCULENS Ophthalmology Clinic, 400347 Cluj-Napoca, Romania; radupop1985@yahoo.com (R.-N.P.); nicula_patricia@yahoo.com (P.N.); niculacristina65@yahoo.com (C.N.); niculadorin60@yahoo.com (D.N.); 2 Faculty of Medicine, “Lucian Blaga” University, 550169 Sibiu, Romania; 3 Department of Oral and Maxillofacial Surgery and Radiology, Faculty of Dentistry, “Iuliu Hațieganu” University of Medicine and Pharmacy, 400012 Cluj-Napoca, Romania; 4 Surgical Department, University of Agricultural Sciences and Veterinary Medicine, 400372 Cluj-Napoca, Romania

**Keywords:** keratoconus, intracorneal ring segments, KeraRing, corneal cross-linking, Pentacam, Corvis ST-derived biomechanics, corneal tomography

## Abstract

Combined intrastromal corneal ring segment implantation with corneal collagen cross-linking (CXL) aims to improve optical regularity and reduce the risk of ectatic progression in selected eyes with keratoconus. In this retrospective, single-center, complete-case longitudinal series, 58 eyes from 40 patients treated at OCULENS Ophthalmology Clinic between 2019 and 2022 were analyzed after same-session KeraRing implantation and conventional epithelium-off CXL, with baseline and 6-, 12-, 24-, and 36-month follow-up data. Outcomes included uncorrected distance visual acuity (UDVA), corrected distance visual acuity (CDVA), thinnest pachymetry, maximum keratometry (Kmax), the Belin/Ambrosio enhanced ectasia total deviation index (BAD-D), the C and D components of the ABCD classification, and Corvis ST-derived dynamic deformation parameters. Longitudinal continuous outcomes were analyzed as eye-level repeated-measures estimates with Holm-adjusted paired comparisons, and stage distributions were analyzed with the Friedman test. At 36 months, UDVA improved from 0.560 ± 0.151 to 0.469 ± 0.136 logMAR and CDVA from 0.350 ± 0.109 to 0.287 ± 0.092 logMAR (both *p* < 0.001). Kmax decreased from 56.11 ± 3.17 D to 54.87 ± 2.86 D, BAD-D improved from 5.62 ± 1.89 to 4.70 ± 1.75, and thinnest pachymetry measured 440.7 ± 21.9 µm at 36 months, corresponding to 97.0% of baseline thickness. Corvis ST-derived deformation behavior shifted toward lower deformation amplitude and higher stiffness parameter at first applanation (SP-A1), but these device-derived parameters should be interpreted as indirect deformation response metrics rather than as direct tissue stiffness measurements. Two clinically significant complications were recorded (2/58 eyes, 3.4%): one sterile corneal infiltrate requiring ring explantation and one case of corneal melting requiring ring explantation and referral for keratoplasty. The findings support sustained 36-month visual, tomographic, and Corvis ST-derived changes after combined treatment, while interpretation remains limited by the retrospective complete-case design, unavailable denominator for cases excluded because of incomplete follow-up, eye-level analysis with bilateral cases, lack of a control group, and indirect nature of Corvis ST-derived biomechanical assessment.

## 1. Introduction

Keratoconus is a progressive ectatic corneal disorder characterized by stromal thinning, protrusion, irregular astigmatism, and gradual visual decline [1,2]. Once the disease substantially impairs optical quality, treatment goals become twofold: to improve functional vision and to reduce the likelihood of further ectatic decompensation.

Intrastromal corneal ring segments (ICRS) were introduced as a minimally invasive means of flattening and regularizing the cornea, thereby reducing irregular astigmatism and improving visual performance in selected eyes [3,4]. Their principal effect is the geometric redistribution of corneal curvature rather than the direct arrest of ectatic progression. By contrast, epithelium-off corneal collagen cross-linking (CXL) is intended to increase stromal resistance through riboflavin/ultraviolet-A photochemical cross-links, and long-term series have demonstrated durable ectasia control in progressive disease [5,6,7]. Combining ICRS and CXL is therefore clinically appealing when the same eye requires both optical regularization and stabilization, but the contribution of each component cannot be separated without an appropriate comparison group.

Modern keratoconus assessment extends beyond anterior curvature alone. Scheimpflug tomography, the Belin/Ambrosio enhanced ectasia total deviation index (BAD-D), ABCD staging, and dynamic corneal deformation imaging with Corvis ST have been used to characterize geometric, pachymetric, visual-function, and tissue-response domains [8,9,10,11,12]. Corvis ST parameters are indirect in vivo markers of dynamic deformation behavior rather than direct ex vivo measurements of tissue stiffness, and they can be influenced by corneal thickness, corneal geometry, and intraocular pressure. This distinction is particularly important after combined procedures, because ring-induced geometric redistribution and CXL-induced tissue changes occur in the same postoperative period.

Several studies have evaluated visual, refractive, and tomographic outcomes after ICRS combined with CXL [13,14,15,16,17,18,19,20]. In addition, Corvis ST has been used to assess keratoconus biomechanics and postoperative changes after CXL, including parameters such as deformation amplitude, applanation timing, highest-concavity radius, and stiffness parameter at first applanation [9,10,11,12]. Recent reliability work also shows that successive Corvis ST measurements in treated and untreated keratoconus eyes must be interpreted in light of measurement variability and disease severity [12]. Thus, the current literature already supports the relevance of biomechanical assessment; the remaining gap is not whether Corvis ST can detect postoperative deformation changes, but how a same-session KeraRing plus conventional epithelium-off CXL cohort behaves across visual, tomographic, pachymetric, ABCD C/D, and Corvis ST-derived domains over a standardized 36-month follow-up.

The analytical framework of the present study was therefore descriptive and domain-based. First, optical and tomographic regularization were evaluated using UDVA, CDVA, Kmax, and BAD-D. Second, pachymetric and visual-function staging were summarized using the C and D components of the ABCD classification. Third, dynamic deformation response was assessed with selected Corvis ST parameters. Finally, exploratory cross-domain correlations examined whether 36-month changes in tomographic or visual variables were associated with 36-month changes in Corvis ST variables. These correlations were prespecified as hypothesis-generating associations and were not intended to prove causality or a direct mechanistic link.

The ABCD classification ordinarily integrates A, the anterior radius of curvature at the 3 mm zone centered on the thinnest point; B, the posterior radius of curvature in the same zone; C, thinnest pachymetry; and D, best-corrected distance visual acuity [21,22]. Because the retrospective export available for this study did not consistently preserve all A and B values, only C and D could be analyzed longitudinally. This limitation weakens comprehensive ectasia staging and is acknowledged from the outset; C and D are used here only as descriptive pachymetric and visual-function context, not as a substitute for complete ABCD staging.

The aim of this study was to describe the 36-month visual, tomographic, ABCD C/D, and Corvis ST-derived course after combined KeraRing implantation and conventional epithelium-off CXL for keratoconus, while explicitly recognizing the limitations imposed by the retrospective complete-case design, eye-level analysis with bilateral cases, absence of a control group, and indirect nature of Corvis ST-derived biomechanical interpretation.

## 2. Materials and Methods

### 2.1. Study Design

This retrospective, single-center, longitudinal complete-case series was based on eyes treated at OCULENS Ophthalmology Clinic between 2019 and 2022 and reviewed from the institutional clinical database. The database contained standardized preoperative and postoperative measurements for eyes treated with combined KeraRing implantation and conventional epithelium-off corneal collagen cross-linking. Clinical, tomographic, Corvis ST-derived deformation, procedural, and adverse-event data were reviewed retrospectively. All analyses were based on anonymized records extracted from the institutional database. This study was designed to describe longitudinal outcomes after the combined procedure; it was not designed to compare combined treatment with CXL alone, ICRS alone, staged treatment, or untreated keratoconus eyes.

### 2.2. Participants and Study Cohort

**Scheme 1:**
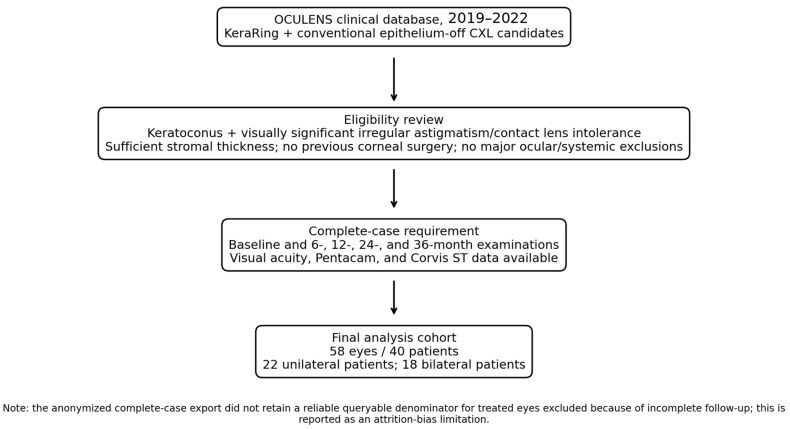
Patient selection and complete-case structure. The flowchart documents the final analyzable cohort and explicitly reports that the anonymized complete-case export did not retain a reliable denominator for all treated eyes excluded because of incomplete scheduled follow-up.

The longitudinal analysis included 58 eyes from 40 patients with complete records at baseline and at 6, 12, 24, and 36 months. Mean age at treatment was 27.2 ± 7.0 years (range, 15–43 years), and 42 eyes (72.4%) belonged to male patients. Twenty-two patients contributed one eye and 18 contributed both eyes. Forty-three eyes (74.1%) received one ring segment and 15 eyes (25.9%) received two segments. The mean ring thickness was 0.24 ± 0.07 mm (range, 0.15–0.35 mm). The anonymized complete-case export used for this revision did not retain a reliable queryable denominator for all eyes initially treated during the study period or for treated eyes excluded because of incomplete scheduled follow-up. Consequently, attrition bias cannot be quantified, and the final cohort should be interpreted strictly as a complete-case longitudinal cohort rather than as all treated eyes during 2019–2022 (Scheme 1).

### 2.3. Inclusion Criteria

Eyes were considered eligible for combined intracorneal ring segment implantation and corneal collagen cross-linking when a clinical and tomographic diagnosis of keratoconus was present together with visually significant irregular astigmatism and/or contact lens intolerance. Additional inclusion requirements were sufficient corneal thickness to permit both stromal tunnel creation and subsequent conventional epithelium-off CXL, a clear central cornea or only minimal paracentral haze, absence of previous corneal surgery in the study eye, and availability of complete baseline and postoperative examinations at 6, 12, 24, and 36 months. Eyes scheduled for cross-linking also had to satisfy the usual intraoperative safety threshold for conventional treatment after epithelial removal and riboflavin saturation.

### 2.4. Exclusion Criteria

Eyes were excluded when advanced corneal scarring involved the visual axis, when previous hydrops had occurred, or when active ocular surface disease, ocular inflammation, clinically relevant endothelial compromise, or severe dry eye could interfere with postoperative healing or data reliability. Additional exclusion criteria were prior corneal surgical procedures, stromal thickness inadequate for safe performance of conventional CXL, pregnancy or breastfeeding at the time of treatment, autoimmune or collagen vascular disease, and any other local or systemic condition considered capable of adversely affecting epithelial recovery, stromal remodeling, or long-term follow-up.

### 2.5. Clinical, Tomographic, and Biomechanical Assessment

All eyes underwent a standardized preoperative and follow-up ophthalmic examination that included uncorrected distance visual acuity (UDVA) and corrected distance visual acuity (CDVA) expressed in logMAR units, manifest refraction, keratometry, slit-lamp biomicroscopy, intraocular pressure assessment, corneal imaging, and specular microscopy. Scheimpflug tomography with Pentacam (Oculus, Wetzlar, Germany) was used to evaluate anterior and posterior corneal elevation, keratometric parameters, pachymetric distribution, thinnest corneal thickness, cone morphology, and the Belin/Ambrosio enhanced ectasia total deviation index (BAD-D). Dynamic biomechanical assessment was performed with Corvis ST (Oculus, Wetzlar, Germany), which records corneal deformation during a calibrated air puff using ultra-high-speed Scheimpflug imaging. Parameters retained for longitudinal analysis were first applanation time (A1T), first applanation velocity (A1V), deformation amplitude (DA), peak distance (PD), highest concavity radius (HCR), second applanation time (A2T), second applanation velocity (A2V), and stiffness parameter at first applanation (SP-A1). Only technically reliable measurements were accepted for analysis. Biomechanical findings were interpreted together with tomographic data for surgical planning and postoperative assessment.

The ABCD classification was reviewed when sufficient data were available. In the full ABCD system, A represents the anterior radius of curvature in the 3 mm zone centered on the thinnest point, B represents the posterior radius of curvature in the same zone, C represents thinnest pachymetry, and D represents best-corrected distance visual acuity [21,22]. Because the retrospective dataset did not consistently preserve the complete anterior and posterior radius variables required for A and B staging across all visits, only the C and D components could be analyzed longitudinally. These C and D results were therefore treated as descriptive thickness and corrected-visual-function indicators, not as complete ABCD staging or definitive ectasia stage migration.

### 2.6. Surgical Planning and Procedure

All implanted segments were KeraRing devices (Mediphacos, Belo Horizonte, Brazil). Preoperative planning was based on corneal tomography, manifest refraction, corrected distance visual acuity, cone location, corneal asymmetry, and pachymetry at the intended channel site, in accordance with the KeraRing nomogram supplied by the manufacturer and published nomogram-based planning principles [23,24,25]. In practical terms, the Pentacam sagittal and axial curvature maps were reviewed to classify the ectatic morphology according to cone centralization, the symmetry of the astigmatic lobes, and the relationship between the refractive, topographic, and coma axes. Pattern-oriented classifications used in the current KeraRing literature describe phenotypes such as croissant, duck, snowman, nipple, and bow-tie [23]. Central or relatively symmetric cones are generally considered more suitable for symmetric segment implantation, frequently with two segments, in order to obtain a more homogeneous flattening effect, whereas inferior, decentered, or markedly asymmetric cones are more often managed with a single segment or an asymmetric configuration targeted at the steeper hemicornea [23,24].

Within this framework, croissant and bow-tie patterns usually favor symmetric segments, snowman phenotypes may require either symmetric long-arc or asymmetric correction depending on subtype and cone centration, and duck-type ectasia more commonly requires an asymmetric implant strategy aligned with the inferior or paracentral cone [23]. The final choice of the number of segments, arc length, thickness, and implantation meridian was individualized according to mean and steep keratometry, refractive cylinder, spherical equivalent, cone eccentricity, and stromal safety. In asymmetric cones, the treatment was directed toward the steeper hemicornea and principal axis of ectasia, whereas in more central cones the goal was to distribute flattening more evenly across the optical zone [23,24,25]. The published nomogram literature supports the concept that greater flattening is obtained with thicker segments and smaller implantation diameters, which is consistent with the 5 to 6 mm channel geometry adopted in the present series [25,26]. Recent analyses of asymmetric ring-segment geometry and epithelial remodeling further support individualized planning in markedly asymmetric cones [27].

The stromal tunnel was created with a Zeiss VisuMax 500 femtosecond laser (Carl Zeiss Meditec, Jena, Germany) at approximately 75% to 80% of the local corneal thickness. In all eyes, the inner portion of the ring channel corresponded to the 5 mm corneal diameter and the outer portion to the 6 mm corneal diameter. After tunnel creation, one or two ring segments were implanted and centered within the prepared intrastromal channel according to the planned meridian. Conventional epithelium-off CXL was subsequently performed in the same eye after ring implantation. Following topical anesthesia, the central corneal epithelium was removed over an area of approximately 9 mm, after which standard riboflavin 0.1% with 20% dextran (Peschke D, Peschke-MediTrade GmbH, Hünenberg, Switzerland) was instilled every 3 min for 30 min. Ultraviolet-A irradiation was then delivered using a Peschke D UVA lamp (Peschke-MediTrade GmbH, Hünenberg, Switzerland) at 3 mW/cm^2^ for 30 min, corresponding to a total fluence of 5.4 J/cm^2^, with repeated riboflavin application at regular intervals throughout exposure. At the end of the procedure, antibiotic and corticosteroid drops were administered, and a bandage contact lens was placed until complete epithelial healing was achieved [5].

### 2.7. Statistical Analysis

Continuous variables are presented as mean ± standard deviation. Longitudinal change across the five study visits was analyzed at the eye level with repeated-measures analysis of variance (RM-ANOVA). The RM-ANOVA *p* values in the tables refer to the overall time effect across baseline and 6-, 12-, 24-, and 36-month visits. To define the timing of postoperative change relative to baseline, paired *t* tests were performed for each postoperative visit with Holm adjustment for multiple comparisons. The C- and D-stage distributions were evaluated with the Friedman test, for which *p* values refer to longitudinal changes in stage distribution. Exploratory Pearson correlation analyses assessed relationships between 36-month change in Kmax and 36-month change in SP-A1, between 36-month change in CDVA and 36-month change in DA, and between 36-month change in BAD-D and 36-month change in Kmax. These correlation *p* values are exploratory eye-level values and were not used to infer causality. A *p* value < 0.05 was considered statistically significant. Statistical analyses were performed using R software, version 4.3.3 (R Foundation for Statistical Computing, Vienna, Austria). Descriptive 95% confidence intervals were calculated for key baseline and 36-month means as mean ± 1.96 standard error. Because individual paired covariance data and inter-eye clustering information were not available in the final anonymized summary export, these confidence intervals are descriptive CIs for means rather than mixed-effects CIs or paired-change CIs.

The analysis has two important statistical limitations. First, 18 patients contributed both eyes, but inter-eye correlation was not modeled; therefore, all *p* values should be interpreted as eye-level estimates that may be overly optimistic. Mixed-effects models or generalized estimating equations would have been preferable if patient-level clustering and raw longitudinal data had been available. Second, residual-level diagnostics for normality, formal sphericity testing, and Greenhouse–Geisser correction could not be reconstructed from the available anonymized summary export. The RM-ANOVA results are therefore retained as descriptive longitudinal estimates and are interpreted cautiously throughout the Results and Discussion.

### 2.8. Ethics

This study adhered to the tenets of the Declaration of Helsinki and to institutional regulations governing research involving human participants. Ethical approval was obtained from the Ethics Committee of OCULENS Ophthalmology Clinic (approval No. 5, dated 2 April 2026). Written informed consent had been obtained before treatment and for anonymized use of clinical, tomographic, and biomechanical data for research purposes; for patients younger than 18 years, consent was obtained from a parent or legal guardian, with patient assent when applicable.

## 3. Results

### 3.1. Cohort Profile

The final complete-case study cohort comprised 58 eyes from 40 patients with scheduled follow-up through 36 months. Twenty-two patients contributed one eye and 18 contributed both eyes. Forty-three eyes (74.1%) received one ring segment and 15 eyes (25.9%) received two segments. The mean ring thickness was 0.24 ± 0.07 mm (range, 0.15–0.35 mm). Because the available anonymized export did not retain the total number of eyes initially treated or excluded because of incomplete scheduled follow-up, selection and attrition bias cannot be quantified. Baseline and procedural characteristics are summarized in Table 1.

### 3.2. Visual, Tomographic, and BAD-D Outcomes

**Figure 1:**
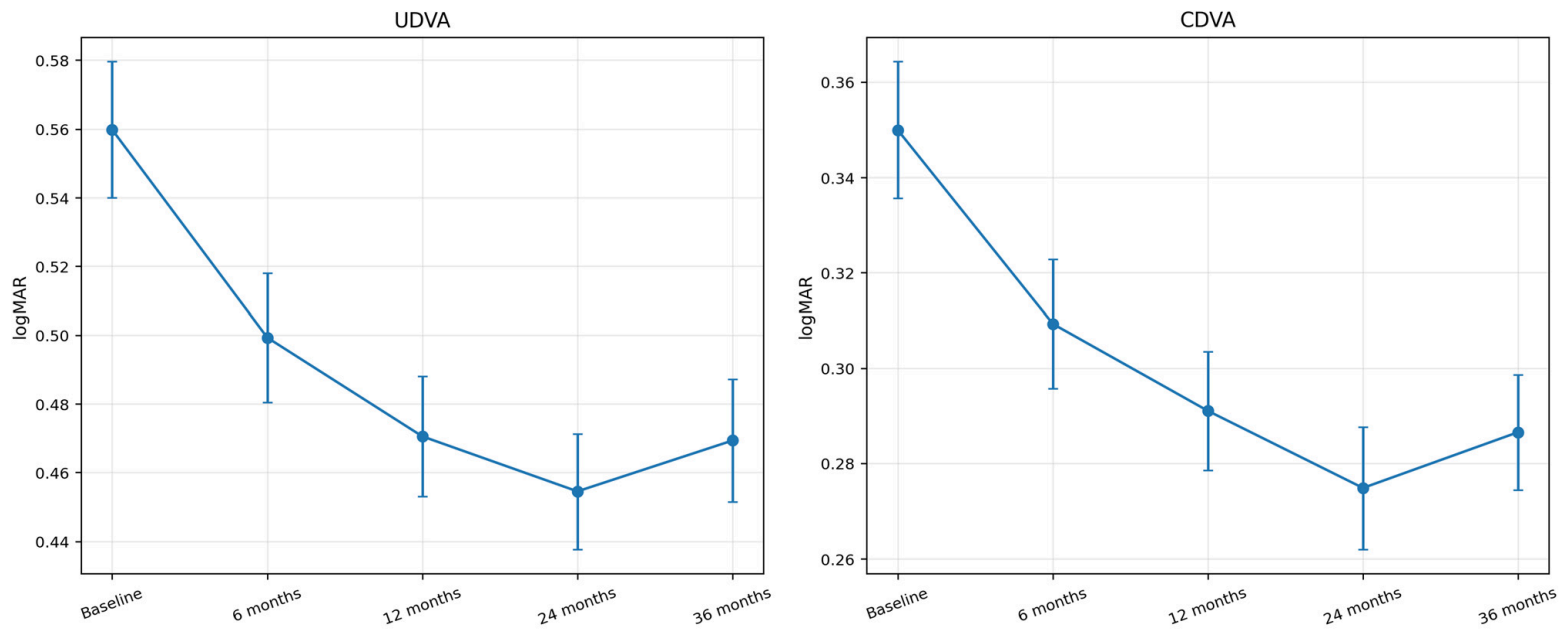
UDVA and CDVA over 36 months after combined KeraRing implantation and conventional epithelium-off CXL. Points represent cohort means and error bars indicate standard errors of the mean. Blue circular markers connected by blue lines identify the plotted mean values. Lower logMAR values indicate better visual acuity. Overall eye-level RM-ANOVA time effects were significant for both UDVA and CDVA (Table 2); Holm-adjusted baseline-versus-follow-up *p* values are provided in Table A1.

**Figure 2:**
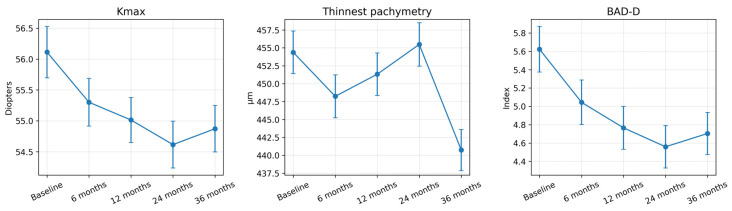
Kmax, thinnest pachymetry, and BAD-D over 36 months. Points represent means and error bars indicate standard errors of the mean. Blue circular markers connected by blue lines identify the plotted mean values. Kmax and BAD-D decreased significantly over time, whereas pachymetry showed early thinning, partial 24-month recovery, and a lower 36-month value. Overall eye-level RM-ANOVA *p* values are shown in Table 2, and Holm-adjusted baseline-versus-follow-up *p* values are shown in Table A1.

Both visual acuity measures improved significantly over time (Table 2; Figure 1 and Figure 2). Mean UDVA improved from 0.560 ± 0.151 logMAR at baseline to 0.454 ± 0.128 at 24 months and remained better than the baseline at 0.469 ± 0.136 at 36 months (overall *F*(4, 228) = 125.01, *p* < 0.001). Mean CDVA improved from 0.350 ± 0.109 to 0.275 ± 0.098 at 24 months and was 0.287 ± 0.092 at 36 months (overall *F*(4, 228) = 116.02, *p* < 0.001). All postoperative comparisons versus baseline remained significant after Holm correction for both UDVA and CDVA (Table A1).

Tomographically, the greatest flattening was observed during the first two postoperative years, while pachymetric change followed a separate remodeling pattern. Mean Kmax decreased from 56.11 ± 3.17 D at baseline to 54.62 ± 2.89 D at 24 months and remained lower than baseline at 54.87 ± 2.86 D at 36 months (overall *F*(4, 228) = 96.29, *p* < 0.001). Thinnest pachymetry showed an early postoperative decrease from 454.4 ± 22.6 µm at baseline to 448.2 ± 22.8 µm at 6 months, followed by partial recovery to 455.5 ± 23.1 µm at 24 months and a final decrease to 440.7 ± 21.9 µm at 36 months. The final 36-month value represented 97.0% of the baseline mean pachymetry (440.7/454.4 × 100 = 97.0%), corresponding to a mean reduction of 13.6 µm. After Holm correction, the 6-, 12-, and 36-month pachymetry values were significantly different from baseline, whereas the 24-month value was not significantly different from baseline (Table A1).

BAD-D improved steadily throughout follow-up, decreasing from 5.62 ± 1.89 at baseline to 4.56 ± 1.77 at 24 months and 4.70 ± 1.75 at 36 months (overall *F*(4, 228) = 260.72, *p* < 0.001). All postoperative BAD-D comparisons remained significant after Holm correction.

The mean 36-month Kmax reduction was 1.24 D. This change reached statistical significance, but its magnitude should be interpreted as a modest, rather than a large, flattening effect. Similarly, the 36-month CDVA improvement of 0.063 logMAR corresponds approximately to three ETDRS letters. This is a small mean gain at cohort level, although it may still be clinically useful in selected keratoconus eyes when combined with improved corneal regularity, stabilization of ectatic progression, and reduced irregular astigmatism.

The 36-month Corvis ST changes were statistically significant across the included parameters; however, the clinical magnitude of these device-derived changes should be interpreted cautiously because universally accepted minimal clinically important differences for postoperative keratoconus follow-up are not established. Corvis ST parameters are also affected by corneal thickness, geometry, and intraocular pressure, not only by intrinsic stromal stiffness.

### 3.3. Corvis ST Biomechanical Outcomes

**Figure 3:**
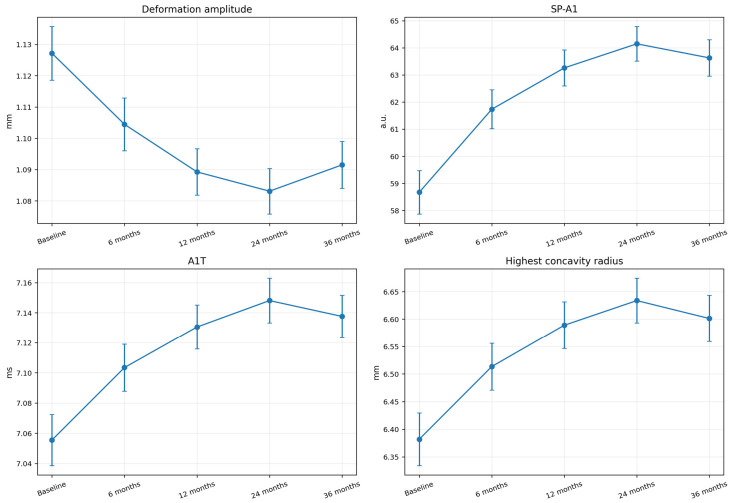
Key Corvis ST-derived deformation outcomes over 36 months: deformation amplitude, SP-A1, A1T, and highest concavity radius. Points represent means and error bars indicate standard errors of the mean. Blue circular markers connected by blue lines identify the plotted mean values. Overall eye-level RM-ANOVA *p* values for these variables were <0.001 (Table 3); these device-derived parameters should be interpreted as deformation response metrics influenced by corneal geometry, thickness, and intraocular pressure.

**Figure 4:**
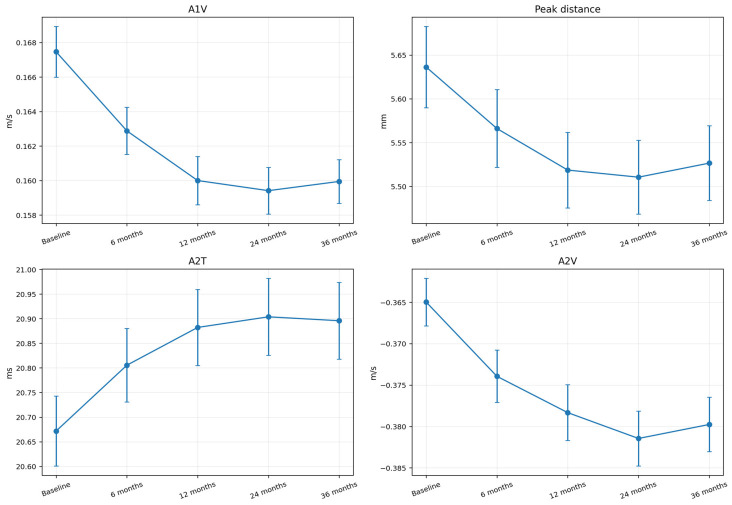
Additional Corvis ST-derived deformation outcomes over 36 months: A1V, peak distance, A2T, and A2V. Points represent means and error bars indicate standard errors of the mean. Blue circular markers connected by blue lines identify the plotted mean values. Overall eye-level RM-ANOVA *p* values were <0.001 for all displayed variables (Table 3), and Holm-adjusted baseline-versus-follow-up *p* values are provided in Table A2.

The Corvis ST-derived deformation profile changed in a direction compatible with reduced postoperative dynamic deformation (Table 3; Figure 3 and Figure 4). Mean DA decreased from 1.127 ± 0.065 mm at baseline to 1.091 ± 0.057 mm at 36 months, whereas mean SP-A1 increased from 58.67 ± 6.10 to 63.63 ± 5.10 (both overall eye-level RM-ANOVA *p* < 0.001). A1T and A2T increased over time, A1V decreased, A2V became more negative, PD decreased, and HCR increased. These findings should be interpreted as indirect changes in the Corvis ST-derived deformation response rather than as direct measurements of intrinsic tissue stiffness. Every postoperative comparison versus baseline remained significant after Holm adjustment for all Corvis ST variables included in the analysis (Table A2).

### 3.4. C- and D-Stage Distributions

**Figure 5:**
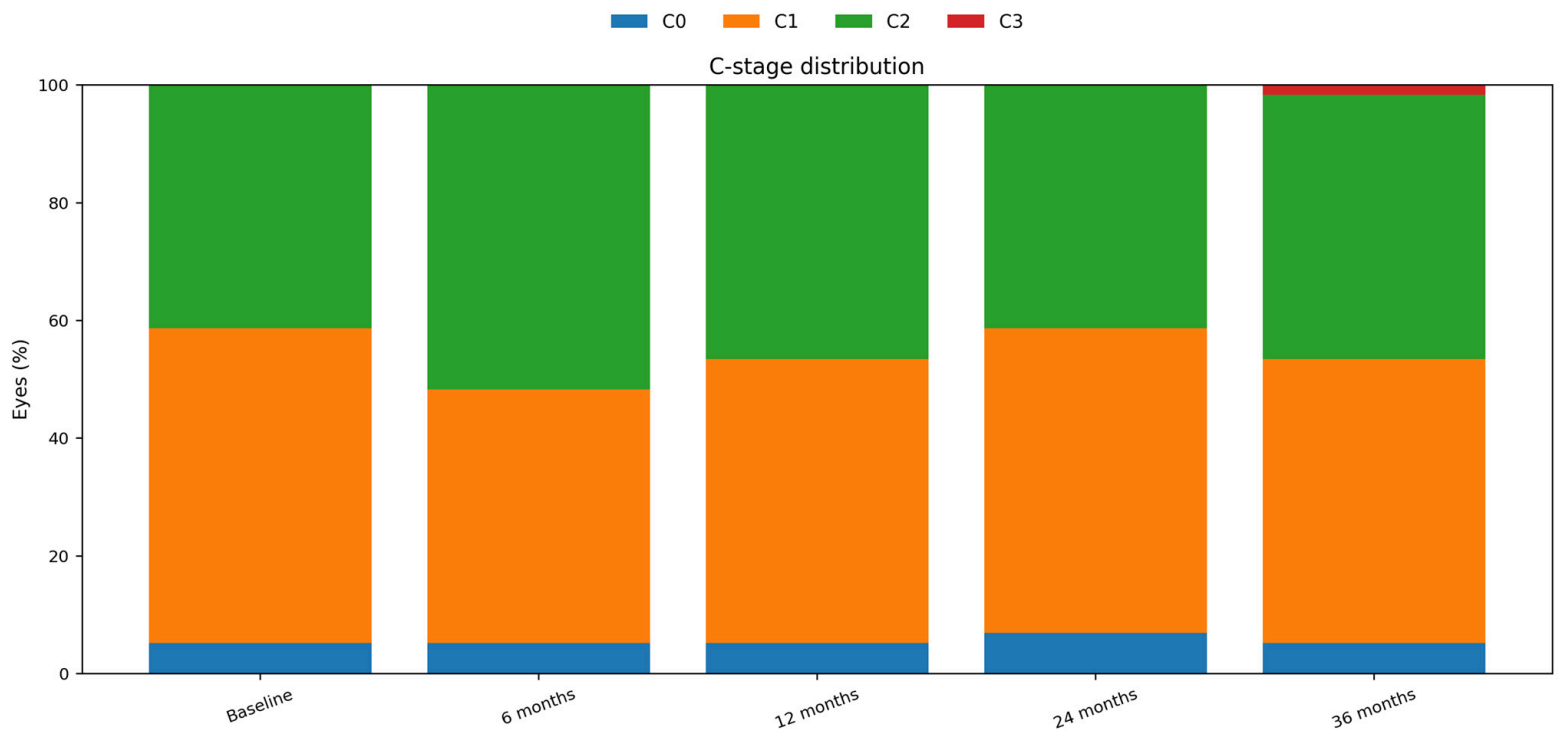
C-component distribution over time. Bars show the percentage of eyes in each pachymetric C category at each visit. The Friedman *p* value shown in Table 4 refers to longitudinal change in C-stage distribution and should be interpreted as a descriptive thickness-domain result rather than as complete ABCD staging.

**Figure 6:**
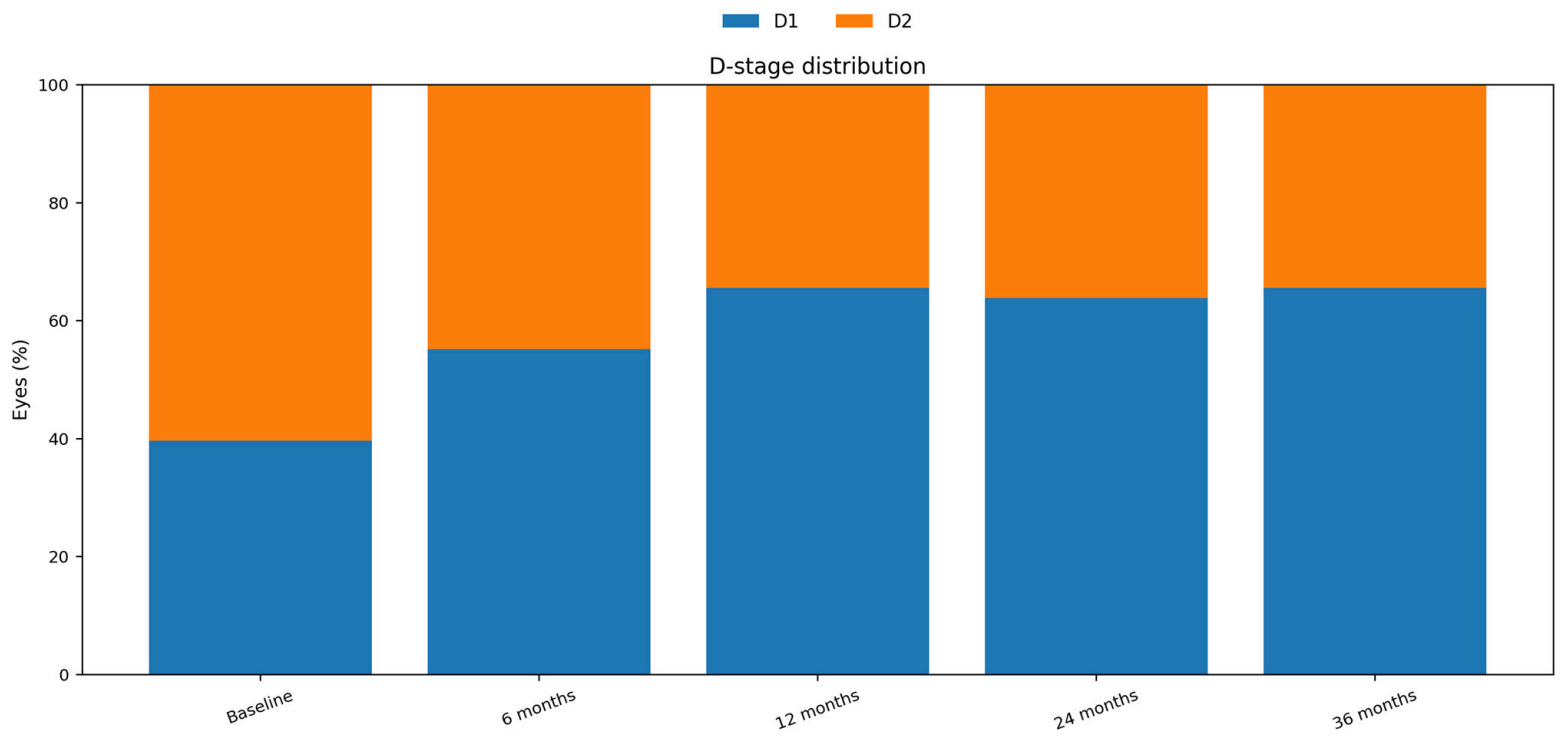
D-component distribution over time. Bars show the percentage of eyes in each corrected visual acuity D category at each visit. The Friedman *p* value shown in Table 5 refers to longitudinal change in D-stage distribution and should be interpreted as a visual-function-domain result, not complete ABCD staging.

C- and D-stage distributions are summarized in Table 4 and Table 5 and illustrated in Figure 5 and Figure 6. Because complete A and B data were unavailable, these results should not be interpreted as complete ABCD stage migration. The C-stage profile showed a modest longitudinal shift in pachymetric category (Friedman *χ*^2^ = 11.45, *p* = 0.022). C2 eyes accounted for 41.4% of the cohort at baseline, increased to 51.7% at 6 months, returned to 41.4% at 24 months, and measured 44.8% at 36 months, with one eye classified as C3 at the final visit. The D-stage profile improved more clearly over time (Friedman *χ*^2^ = 43.47, *p* < 0.001): D1 eyes increased from 39.7% at baseline to 65.5% at 12 months and remained at 65.5% at 36 months.

### 3.5. Structure–Function Correlations

At the eye level, 36-month flattening in Kmax correlated with a 36-month increase in SP-A1 (*r* = −0.539, *p* < 0.001), improvement in CDVA correlated with reduction in DA (*r* = 0.423, *p* < 0.001), and improvement in BAD-D correlated with flattening in Kmax (*r* = 0.372, *p* = 0.004). These correlations were moderate, exploratory, and calculated at the eye level without adjustment for bilateral cases; therefore, they show co-occurrence of favorable changes but do not establish causality or a mechanistic link between tomographic regularization and Corvis ST-derived deformation response.

### 3.6. Representative Pentacam Case Examples

Representative Pentacam examples were removed from the main Results section and placed in Supplementary Figures S1–S8. They are retained only as illustrative examples of individual tomographic remodeling and do not replace the cohort-level analyses in Table 1, Table 2, Table 3, Table 4, Table 5, Table 6 and Table 7 and Figure 1, Figure 2, Figure 3, Figure 4, Figure 5, Figure 6 and Figure 7.

### 3.7. Complications

Two eyes experienced clinically significant postoperative complications requiring ring explantation. One eye developed a sterile corneal infiltrate, and a second eye developed corneal melting and was referred for keratoplasty. These events correspond to 3.4% of the 58-eye complete-case cohort. Because the series is small and uncontrolled, this percentage should not be generalized as a population rate; however, it is clinically important and cannot be dismissed. Without ICRS-alone, CXL-alone, staged-procedure, or untreated controls, this study cannot determine whether these events were attributable to the ring procedure, CXL-related healing, ocular-surface or patient-specific risk factors, surgical interaction, or a combination of these mechanisms.

## 4. Discussion

This retrospective single-center complete-case series showed that combined treatment with KeraRing implantation and conventional epithelium-off CXL was associated with sustained visual improvement, tomographic regularization, BAD-D reduction, and Corvis ST-derived deformation changes over 36 months. The clinical rationale for the combined approach is that ICRS can regularize an ectatic cornea and reduce the optical penalty of irregular astigmatism, whereas CXL is intended to reduce the risk of further biomechanical decompensation. The present data support the feasibility of pursuing these goals together in carefully selected eyes, but they do not prove superiority over CXL alone, ICRS alone, staged procedures, or other customized approaches.

The complication profile requires a cautious interpretation. Two clinically significant adverse events requiring ring explantation were observed among 58 eyes (3.4%), including one sterile infiltrate and one case of corneal melting with referral for keratoplasty. Although the small denominator prevents precise safety estimation, this rate is numerically higher than some larger published series and should be explicitly discussed with patients when consent is obtained. In this retrospective uncontrolled design, it is not possible to determine whether the adverse events were inherent to the combined procedure itself, related mainly to the ICRS channel and foreign-body effect, influenced by epithelial removal and wound healing after CXL, or driven by baseline corneal/ocular-surface risk factors. The safest interpretation is therefore descriptive: clinically significant complications were uncommon but meaningful in this cohort and require long-term monitoring. Complications and explantation after ICRS implantation have also been summarized in systematic and incidence-based analyses, which support the need for careful postoperative surveillance [28,29].

The visual and tomographic outcomes are broadly consistent with previous combined-procedure studies [13,14,15,16,17,18,19,20]. They are also consistent with recent comparative and longer-term ICRS literature, including simultaneous accelerated CXL combined with ICRS or alternative customized procedures, systematic review/meta-analysis data, and five-year ICRS follow-up reports [30,31,32]. Mean Kmax decreased by 1.24 D from baseline to 36 months, with the largest flattening observed by 24 months. This magnitude is statistically significant but clinically modest. It should not be presented as a large keratometric effect; rather, it should be understood together with improved BAD-D, improved visual acuity, and qualitative regularization of the anterior curvature profile. Likewise, the mean 36-month CDVA gain of 0.063 logMAR corresponds to approximately three ETDRS letters, which is below the threshold often considered a large visual improvement in clinical trials. The clinical value of the procedure in this cohort therefore lies less in a dramatic mean acuity gain and more in sustained optical regularization, preservation of function, and absence of obvious cohort-level ectatic progression over 36 months. The broader evidence base for epithelium-off CXL, including systematic reviews and randomized trials, supports its role in slowing progressive keratoconus [33,34,35,36].

The pachymetric course deserves careful interpretation. The mean thinnest pachymetry decreased early, partially recovered by 24 months, and then measured 440.7 ± 21.9 µm at 36 months, corresponding to 97.0% of the baseline mean. Several mechanisms may explain this pattern. Early postoperative thinning after epithelium-off CXL may reflect epithelial remodeling, stromal dehydration or compaction, keratocyte/apoptotic and wound-healing changes, and the redistribution of the thinnest point after ring-induced geometric remodeling. Partial recovery by 24 months may reflect epithelial and stromal remodeling toward a new postoperative equilibrium. The lower 36-month value may reflect persistent remodeling, measurement variability at the thinnest point in an irregular cornea, or true residual thinning in some eyes. Therefore, pachymetry should not be interpreted in isolation; stable or slightly reduced pachymetry is reassuring only when accompanied by stable or improved curvature, improved BAD-D, preserved transparency, and absence of progressive posterior elevation.

The BAD-D findings strengthen this contextual interpretation because BAD-D improved significantly throughout follow-up. BAD-D integrates anterior elevation, posterior elevation, pachymetric progression, and relational thickness behavior, so its improvement suggests that postoperative change was not limited to the anterior Kmax value. At the same time, the absence of complete A and B data means that the C and D components cannot be interpreted as complete ABCD stage migration. In this dataset, C-stage findings provide descriptive pachymetric context, and D-stage findings provide corrected-visual-function context only.

The Corvis ST findings should also be interpreted carefully. DA and PD decreased, HCR increased, A1T and A2T lengthened, A1V decreased, A2V became more negative, and SP-A1 increased. Together, these findings are compatible with reduced dynamic deformation under a standardized air puff after combined treatment. They should not be described as direct proof of increased intrinsic stromal stiffness, because Corvis ST parameters are influenced by corneal geometry, thickness, intraocular pressure, hydration, and measurement repeatability. Moreover, minimal clinically important differences for these parameters after combined ICRS and CXL have not been standardized. The most appropriate conclusion is that the Corvis ST-derived profile shifted in a direction compatible with a stiffer postoperative deformation response, not that direct tissue stiffness was measured.

The exploratory correlations provide a cross-domain signal but not mechanistic proof. Greater 36-month flattening in Kmax was associated with a greater increase in SP-A1, and better CDVA was associated with lower DA. These associations are clinically plausible, but they are moderate, eye-level, and unadjusted for bilateral data. They should therefore be interpreted as hypothesis-generating co-occurrences rather than as evidence that tomographic regularization caused biomechanical stiffening or that biomechanical change caused visual improvement.

Methodologically, a comparison with single or staged procedures is not required for a descriptive longitudinal case series, but it is required to determine attribution and comparative effectiveness. A suitable future control group would be a matched CXL-alone cohort with similar baseline severity and documented progression, because this would help separate the stabilizing effect of CXL from the additional optical reshaping effect of ICRS. Other useful designs include ICRS-alone eyes when ethically acceptable, staged ICRS followed by CXL or CXL followed by ICRS cohorts, and carefully selected contralateral-eye comparisons in bilateral disease. Without these groups, the present study can state that outcomes were sustained after combined treatment, but it cannot state that combined treatment was superior to CXL alone or that all biomechanical changes were caused by the combined interaction.

The practical clinical message is deliberately cautious. Combined KeraRing implantation and CXL can be useful in patients whose keratoconus causes both optical irregularity and concern for progression, particularly when spectacles or contact lenses no longer provide satisfactory functional vision. At the same time, it is not a purely refractive procedure, it is not risk-free, and it should not be presented as proven superior to single or staged procedures on the basis of this study alone. Surgical planning should integrate manifest refraction, CDVA, cone morphology, anterior and posterior elevation, thinnest pachymetry, intended channel depth, ocular-surface status, and patient expectations. Long-term follow-up remains necessary because visual improvement, pachymetric remodeling, and Corvis ST-derived deformation changes do not always evolve at the same pace.

Several limitations should be acknowledged. First, this was a retrospective complete-case analysis, and the anonymized export did not preserve a reliable denominator for all treated eyes or eyes excluded because of incomplete follow-up; therefore, attrition bias cannot be quantified. Second, this was an eye-level analysis in which 18 patients contributed both eyes, and inter-eye correlation was not modeled; this is a major statistical limitation and may overstate the apparent precision and statistical significance of the results. Third, residual normality, sphericity testing, and Greenhouse–Geisser correction could not be reconstructed from the available summary export, so RM-ANOVA *p* values should be interpreted cautiously. Fourth, this study was uncontrolled and cannot establish superiority over CXL alone, ICRS alone, or alternative sequencing strategies. Fifth, complete A and B ABCD variables were not consistently available in all records, limiting stage-based analysis to C and D descriptors. Sixth, Corvis ST-derived parameters are indirect deformation metrics rather than direct mechanical measurements of corneal tissue. Seventh, the sample size remains modest for subgroup and safety analyses. Finally, the representative Pentacam cases have been moved to the Supplementary Material because they are illustrative examples and do not replace cohort-level statistics.

Future prospective studies should be designed to address these limitations directly. Mixed-effects models would allow repeated visits to be analyzed while accounting for patient-level clustering when both eyes are included. One-eye sensitivity analyses would test whether results remain similar when only one randomly selected or clinically eligible eye per patient is analyzed. Matched comparison groups would clarify how much of the observed outcome is attributable to ring-induced reshaping, CXL-induced stabilization, natural disease behavior, or a true interaction between ring implantation and cross-linking. Such studies should also prospectively record the full treated-eye denominator, excluded cases, reasons for loss to follow-up, sphericity diagnostics, corrected *p* values, and clinically meaningful thresholds for Corvis ST-derived outcomes.

## 5. Conclusions

Combined treatment with KeraRing implantation and conventional epithelium-off corneal collagen cross-linking was associated with sustained 36-month improvement in UDVA, CDVA, Kmax, BAD-D, and several Corvis ST-derived deformation parameters in this retrospective single-center complete-case series. The D-component improved in parallel with corrected visual acuity, whereas the C-component reflected early thinning, partial 24-month recovery, and a final 36-month pachymetry decrease to approximately 97.0% of baseline. These findings support combined treatment as a potentially useful option in selected keratoconus eyes requiring both optical regularization and stabilization; however, this study does not prove superiority over CXL alone, ICRS alone, or staged treatment. Interpretation is limited by the retrospective complete-case design, unavailable denominator for cases excluded because of incomplete follow-up, eye-level analysis with bilateral cases, lack of control group, incomplete ABCD staging, and indirect nature of Corvis ST-derived biomechanical assessment. Clinically significant complications requiring ring explantation occurred in 2/58 eyes and must be included in patient counseling and long-term postoperative surveillance.

## Figures and Tables

**Figure 7:**
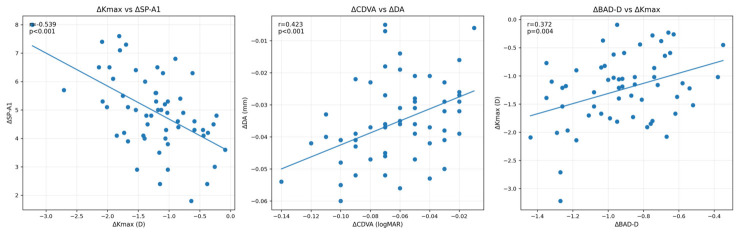
Exploratory 36-month eye-level correlation analyses for ΔKmax versus ΔSP-A1, ΔCDVA versus ΔDA, and ΔBAD-D versus ΔKmax. The plotted regression lines summarize associations only. Blue points represent individual eyes, and blue lines show fitted linear regression trends. These correlations were moderate and unadjusted for bilateral cases; they do not establish causality or a direct mechanism linking tomographic and Corvis ST-derived changes.

**Table 1:** Cohort and procedural characteristics.

Characteristic	Value
Final study cohort, *n*	58
Age at treatment, years	27.2 ± 7.0
Age range, years	15–43
Eyes from male patients, *n* (%)	42 (72.4)
Eyes from female patients, *n* (%)	16 (27.6)
Study years	2019–2022
Source patients, *n*	40
Unilateral patients, *n*	22
Bilateral patients, *n*	18
One ring implanted, *n* (%)	43 (74.1)
Two rings implanted, *n* (%)	15 (25.9)
Ring thickness, mm	0.24 ± 0.07
Ring thickness range, mm	0.15–0.35

Note: All clinical, tomographic, biomechanical, and procedural descriptors were derived from the same 58-eye complete-case cohort. The source denominator for all initially treated eyes was not recoverable from the anonymized export; therefore, excluded cases with incomplete follow-up cannot be compared with included cases.

**Table 2:** Longitudinal visual, tomographic, and BAD-D outcomes.

Variable	Baseline	6 Months	12 Months	24 Months	36 Months	RM-ANOVA
UDVA (logMAR)	0.560 ± 0.151	0.499 ± 0.143	0.471 ± 0.134	0.454 ± 0.128	0.469 ± 0.136	*F*(4, 228) = 125.01; *p* < 0.001; *ηp*^2^ = 0.687
CDVA (logMAR)	0.350 ± 0.109	0.309 ± 0.103	0.291 ± 0.095	0.275 ± 0.098	0.287 ± 0.092	*F*(4, 228) = 116.02; *p* < 0.001; *ηp*^2^ = 0.671
Thinnest pachymetry (µm)	454.4 ± 22.6	448.2 ± 22.8	451.3 ± 22.6	455.5 ± 23.1	440.7 ± 21.9	*F*(4, 228) = 208.90; *p* < 0.001; *ηp*^2^ = 0.786
Kmax (D)	56.11 ± 3.17	55.30 ± 2.94	55.01 ± 2.78	54.62 ± 2.89	54.87 ± 2.86	*F*(4, 228) = 96.29; *p* < 0.001; *ηp*^2^ = 0.628
BAD-D	5.62 ± 1.89	5.04 ± 1.85	4.77 ± 1.79	4.56 ± 1.77	4.70 ± 1.75	*F*(4, 228) = 260.72; *p* < 0.001; *ηp*^2^ = 0.821

Values are mean ± standard deviation. RM-ANOVA = repeated-measures analysis of variance; *ηp*^2^ = partial eta squared. The RM-ANOVA *p* value refers to the overall eye-level time effect across all five visits; Holm-adjusted baseline-versus-follow-up *p* values are provided in Table A1.

**Table 3:** Longitudinal Corvis ST outcomes.

Variable	Baseline	6 Months	12 Months	24 Months	36 Months	RM-ANOVA
A1T (ms)	7.056 ± 0.129	7.103 ± 0.119	7.131 ± 0.111	7.148 ± 0.113	7.138 ± 0.108	*F*(4, 228) = 191.61; *p* < 0.001; *ηp*^2^ = 0.771
A1V (m/s)	0.167 ± 0.011	0.163 ± 0.010	0.160 ± 0.011	0.159 ± 0.010	0.160 ± 0.010	*F*(4, 228) = 86.21; *p* < 0.001; *ηp*^2^ = 0.602
DA (mm)	1.127 ± 0.065	1.104 ± 0.064	1.089 ± 0.056	1.083 ± 0.055	1.091 ± 0.057	*F*(4, 228) = 119.26; *p* < 0.001; *ηp*^2^ = 0.677
PD (mm)	5.636 ± 0.354	5.566 ± 0.339	5.519 ± 0.329	5.511 ± 0.320	5.527 ± 0.325	*F*(4, 228) = 71.97; *p* < 0.001; *ηp*^2^ = 0.558
HCR (mm)	6.382 ± 0.363	6.513 ± 0.323	6.589 ± 0.323	6.634 ± 0.309	6.601 ± 0.320	*F*(4, 228) = 219.37; *p* < 0.001; *ηp*^2^ = 0.794
A2T (ms)	20.672 ± 0.541	20.806 ± 0.569	20.882 ± 0.588	20.904 ± 0.595	20.896 ± 0.595	*F*(4, 228) = 159.87; *p* < 0.001; *ηp*^2^ = 0.737
A2V (m/s)	−0.365 ± 0.022	−0.374 ± 0.024	−0.378 ± 0.026	−0.381 ± 0.025	−0.380 ± 0.025	*F*(4, 228) = 251.32; *p* < 0.001; *ηp*^2^ = 0.815
SP-A1 (a.u.)	58.67 ± 6.10	61.73 ± 5.48	63.26 ± 5.03	64.15 ± 4.87	63.63 ± 5.10	*F*(4, 228) = 497.11; *p* < 0.001; *ηp*^2^ = 0.897

Values are mean ± standard deviation. All overall eye-level RM-ANOVA time effects were significant at *p* < 0.001. These *p* values refer to longitudinal change across all visits and do not account for inter-eye correlation in bilateral cases.

**Table 4:** C-stage distribution over time.

Stage	Baseline	6 Months	12 Months	24 Months	36 Months
C0	3 (5.2%)	3 (5.2%)	3 (5.2%)	4 (6.9%)	3 (5.2%)
C1	31 (53.4%)	25 (43.1%)	28 (48.3%)	30 (51.7%)	28 (48.3%)
C2	24 (41.4%)	30 (51.7%)	27 (46.6%)	24 (41.4%)	26 (44.8%)
C3	0 (0.0%)	0 (0.0%)	0 (0.0%)	0 (0.0%)	1 (1.7%)

Friedman test: *χ*^2^ = 11.45, *p* = 0.022.

**Table 5:** D-stage distribution over time.

Stage	Baseline	6 Months	12 Months	24 Months	36 Months
D1	23 (39.7%)	32 (55.2%)	38 (65.5%)	37 (63.8%)	38 (65.5%)
D2	35 (60.3%)	26 (44.8%)	20 (34.5%)	21 (36.2%)	20 (34.5%)

Friedman test: *χ*^2^ = 43.47, *p* < 0.001.

**Table 6:** Exploratory 36-month correlation analyses.

Comparison	Pearson *r*	*p* Value
ΔKmax vs. ΔSP-A1	−0.539	<0.001
ΔCDVA vs. ΔDA	0.423	<0.001
ΔBAD-D vs. ΔKmax	0.372	0.004

Δ indicates 36-month minus baseline change. Pearson *r* and *p* values are exploratory eye-level estimates and were not adjusted for bilateral cases; they should be read as associations rather than as mechanistic evidence.

**Table 7:** Descriptive 95% confidence intervals and clinical reading of key 36-month outcomes.

Outcome	Baseline Mean (95% CI)	36-Month Mean (95% CI)	Mean Change	Clinical Reading
UDVA (logMAR)	0.560 (0.521 to 0.599)	0.469 (0.434 to 0.504)	−0.091 logMAR	Small-to-moderate mean uncorrected acuity gain; lower logMAR is better.
CDVA (logMAR)	0.350 (0.322 to 0.378)	0.287 (0.263 to 0.311)	−0.063 logMAR	Approximately 3 ETDRS letters; statistically significant but modest.
Kmax (D)	56.110 (55.294 to 56.926)	54.870 (54.134 to 55.606)	−1.24 D	Modest flattening; clinically interpreted with BAD-D and visual function.
BAD-D	5.620 (5.134 to 6.106)	4.700 (4.250 to 5.150)	−0.92	Improvement in integrated tomographic ectasia index.
Thinnest pachymetry (µm)	454.400 (448.584 to 460.216)	440.700 (435.064 to 446.336)	−13.7 µm	97.0% of baseline; interpret with curvature, BAD-D, transparency, and posterior elevation.
DA (mm)	1.127 (1.110 to 1.144)	1.091 (1.076 to 1.106)	−0.036 mm	Device-derived lower deformation; MCID not standardized.
SP-A1 (a.u.)	58.670 (57.100 to 60.240)	63.630 (62.317 to 64.943)	+4.96 a.u.	Device-derived higher stiffness parameter; indirect deformation response.

Note: 95% CIs are descriptive confidence intervals around the baseline and 36-month means calculated from the reported standard deviations and *n* = 58. They are not mixed-effects, clustered, or paired-change CIs because paired covariance and patient-level clustering were not available in the anonymized summary export.

## Data Availability

The anonymized data supporting the findings of this study are available from the corresponding author upon reasonable request, subject to institutional and legal restrictions protecting patient confidentiality.

## Supplementary Materials

The following supporting information can be downloaded at: https://www.mdpi.com/article/10.3390/vision10030042/s1, Figure S1: Representative baseline Pentacam refractive map; Figure S2: Representative 36-month Pentacam refractive map; Figure S3: Representative baseline keratoconus-staging display; Figure S4: Representative 36-month keratoconus-staging display; Figure S5: Representative baseline Belin/Ambrosio enhanced ectasia display; Figure S6: Representative 36-month Belin/Ambrosio enhanced ectasia display; Figure S7: Representative baseline corneal-ring planning/tomography display; Figure S8: Representative 36-month postoperative corneal-ring/tomography display.

## Abbreviations

A1T	First applanation time
A1V	First applanation velocity
A2T	Second applanation time
A2V	Second applanation velocity
ABCD	Keratoconus classification based on anterior radius, posterior radius, corneal thickness, and distance visual acuity
BAD-D	Belin/Ambrosio enhanced ectasia total deviation index
CDVA	Corrected distance visual acuity
CI	Confidence interval
CXL	Corneal collagen cross-linking
DA	Deformation amplitude
HCR	Highest concavity radius
ICRS	Intrastromal corneal ring segment
IOP	Intraocular pressure
Kmax	Maximum keratometry
logMAR	Logarithm of the minimum angle of resolution
MCID	Minimal clinically important difference
PD	Peak distance
RM-ANOVA	Repeated-measures analysis of variance
SP-A1	Stiffness parameter at first applanation
UDVA	Uncorrected distance visual acuity
UVA	Ultraviolet-A

## Appendix A

**Table A1:** Holm-adjusted baseline-versus-follow-up *p* values for clinical variables.

Variable	BL vs. 6 mo	BL vs. 12 mo	BL vs. 24 mo	BL vs. 36 mo
UDVA	<0.001	<0.001	<0.001	<0.001
CDVA	<0.001	<0.001	<0.001	<0.001
Thinnest pachymetry	<0.001	<0.001	0.105	<0.001
Kmax	<0.001	<0.001	<0.001	<0.001
BAD-D	<0.001	<0.001	<0.001	<0.001

Holm-adjusted paired *t*-test *p* values are shown.

**Table A2:** Holm-adjusted baseline-versus-follow-up *p* values for Corvis ST variables.

Variable	BL vs. 6 mo	BL vs. 12 mo	BL vs. 24 mo	BL vs. 36 mo
A1T	<0.001	<0.001	<0.001	<0.001
A1V	<0.001	<0.001	<0.001	<0.001
DA	<0.001	<0.001	<0.001	<0.001
PD	<0.001	<0.001	<0.001	<0.001
HCR	<0.001	<0.001	<0.001	<0.001
A2T	<0.001	<0.001	<0.001	<0.001
A2V	<0.001	<0.001	<0.001	<0.001
SP-A1	<0.001	<0.001	<0.001	<0.001

Holm-adjusted paired *t*-test *p* values are shown.
