# Supplementary material for: Three-Year Visual, Tomographic, and Corvis ST-Derived Biomechanical Outcomes After Combined Intrastromal Ring Implantation and Corneal Collagen Cross-Linking for Keratoconus
Source: Vision (Basel). 2026 Jul 6;10(3):42. doi: 10.3390/vision10030042 (PMC13398343; doi:10.3390/vision10030042)
Supplement: Supplementary file 1 [file vision-10-00042-s001.zip › vision-4339843-supplementary.pdf]

Supplementary Material

Representative serial Pentacam case examples moved from the main manuscript to improve readability. Patient-identifying information was removed/anonymized before inclusion in the manuscript.

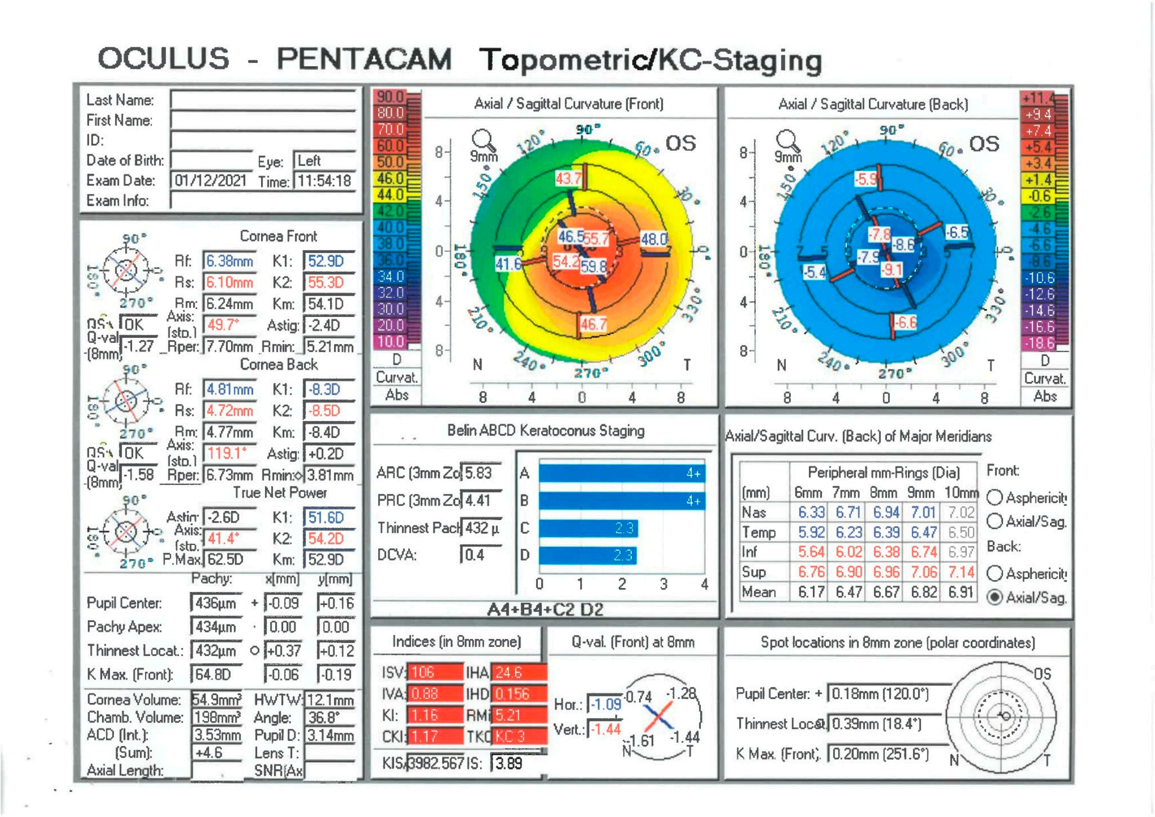

Supplementary Figure S1. Representative unilateral case, left eye: baseline Pentacam Topometric/KC-Staging display obtained on 01 December 2021. Patient-identifying data were removed/anonymized before inclusion in the manuscript.

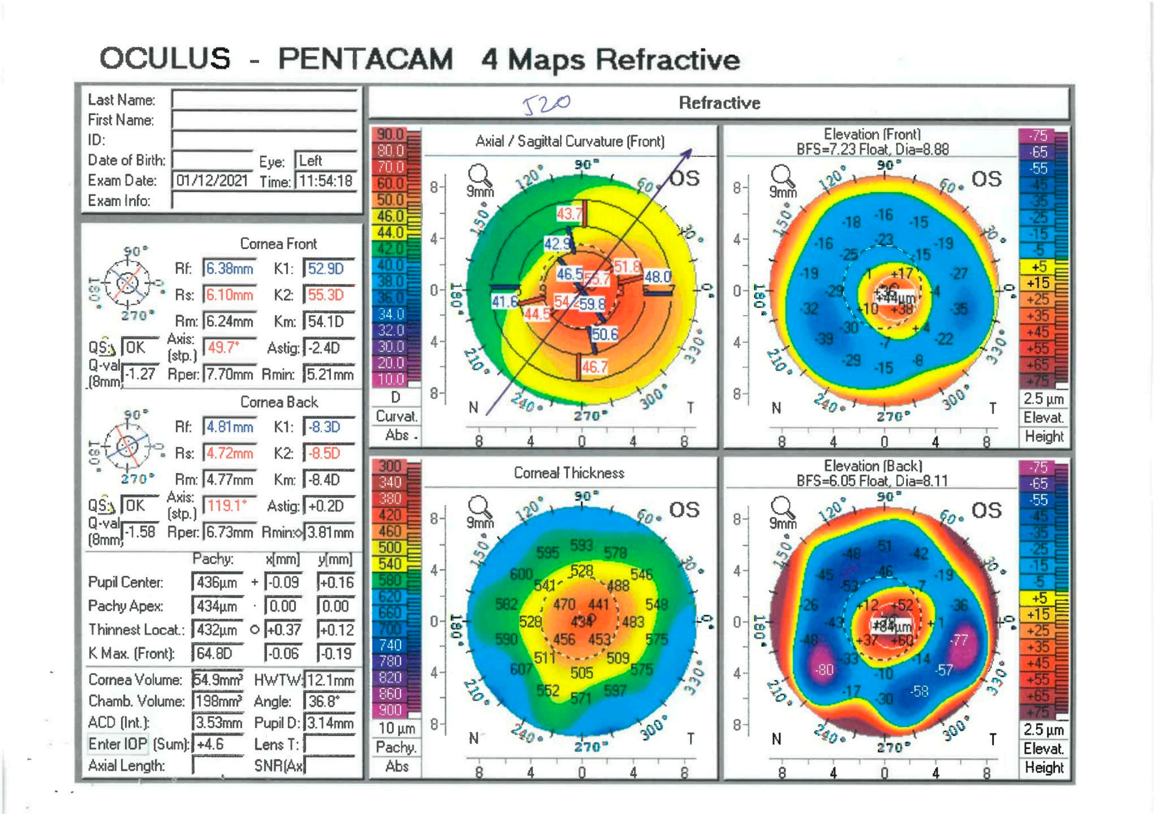

Supplementary Figure S2. Same eye: baseline Pentacam 4 Maps Refractive display showing inferior paracentral steepening and irregular astigmatism before combined KeraRing implantation and CXL.

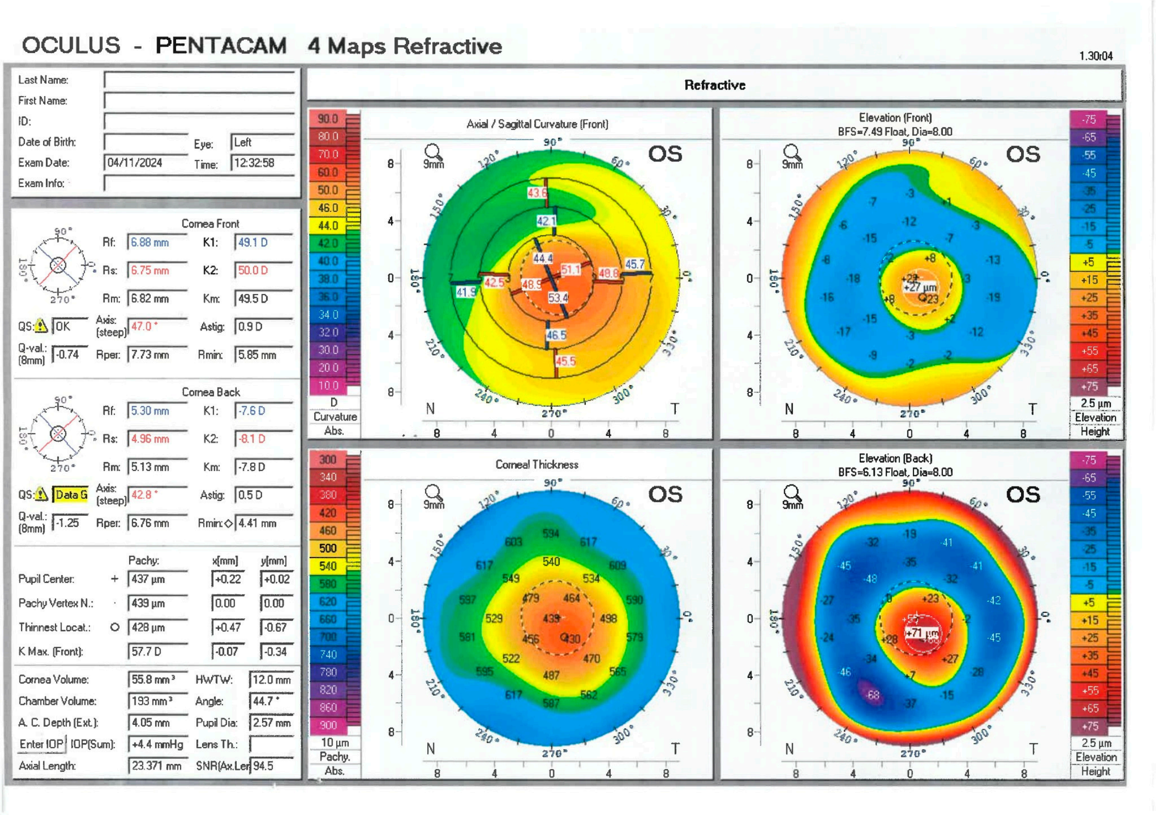

Supplementary Figure S3. Same eye: approximately 36-month postoperative Pentacam 4 Maps Refractive display showing corneal flattening, central regularization of anterior astigmatism, reduced Kmax, and preservation of thinnest pachymetry close to baseline.

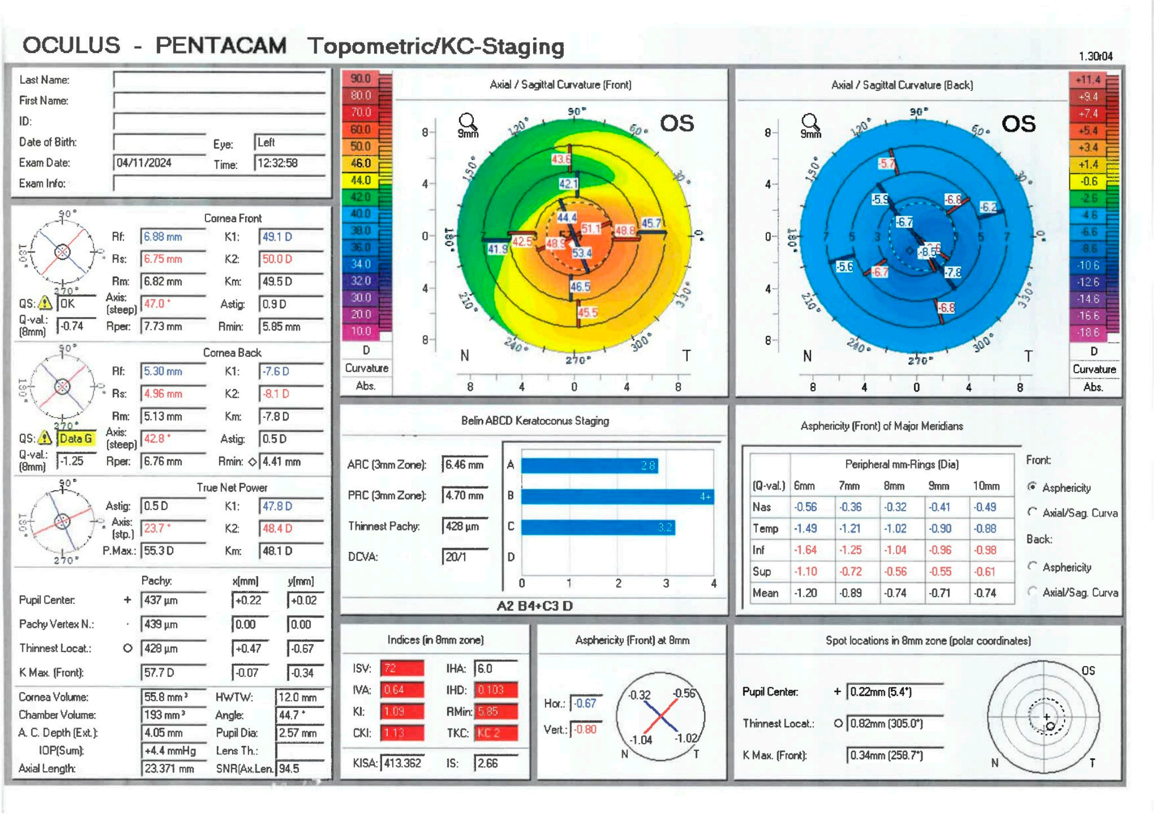

Supplementary Figure S4. Same eye: approximately 36-month postoperative Pentacam Topometric/KC-Staging display confirming a more regular anterior curvature profile.

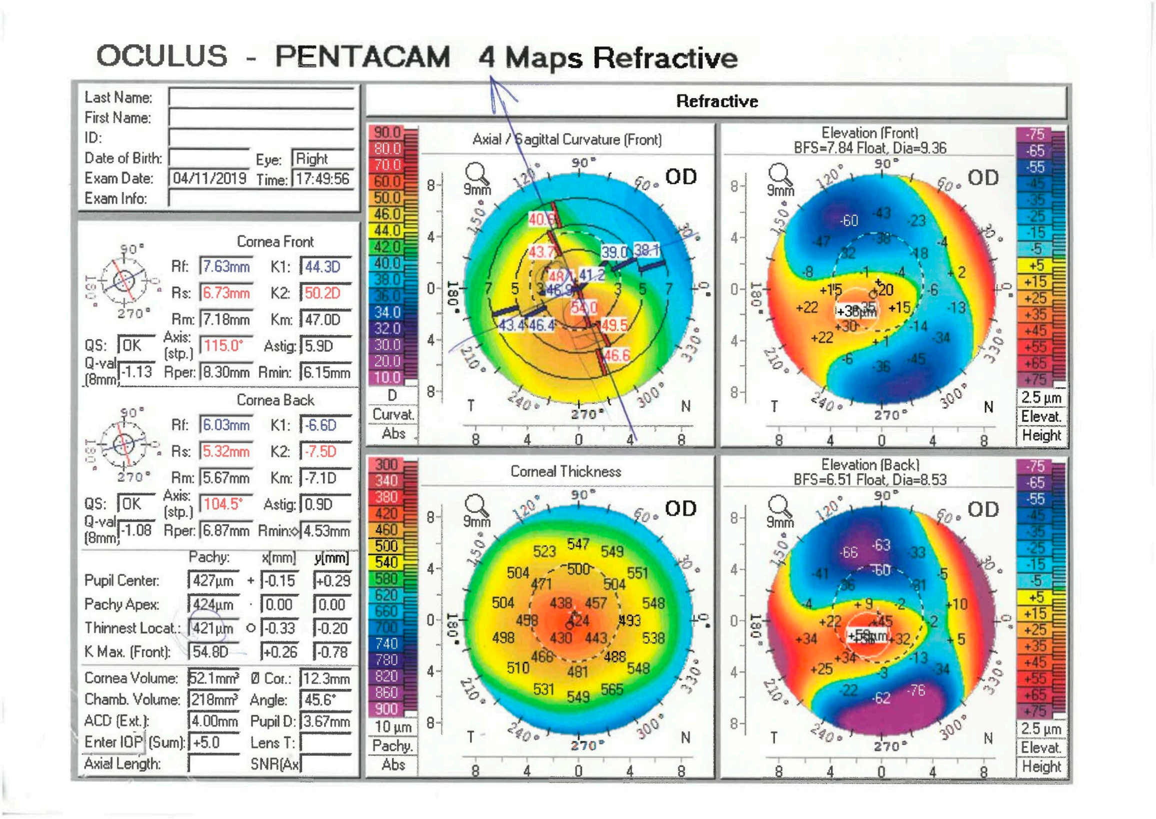

Supplementary Figure S5. Representative bilateral case, right eye: baseline Pentacam 4 Maps Refractive display obtained on 04 November 2019.

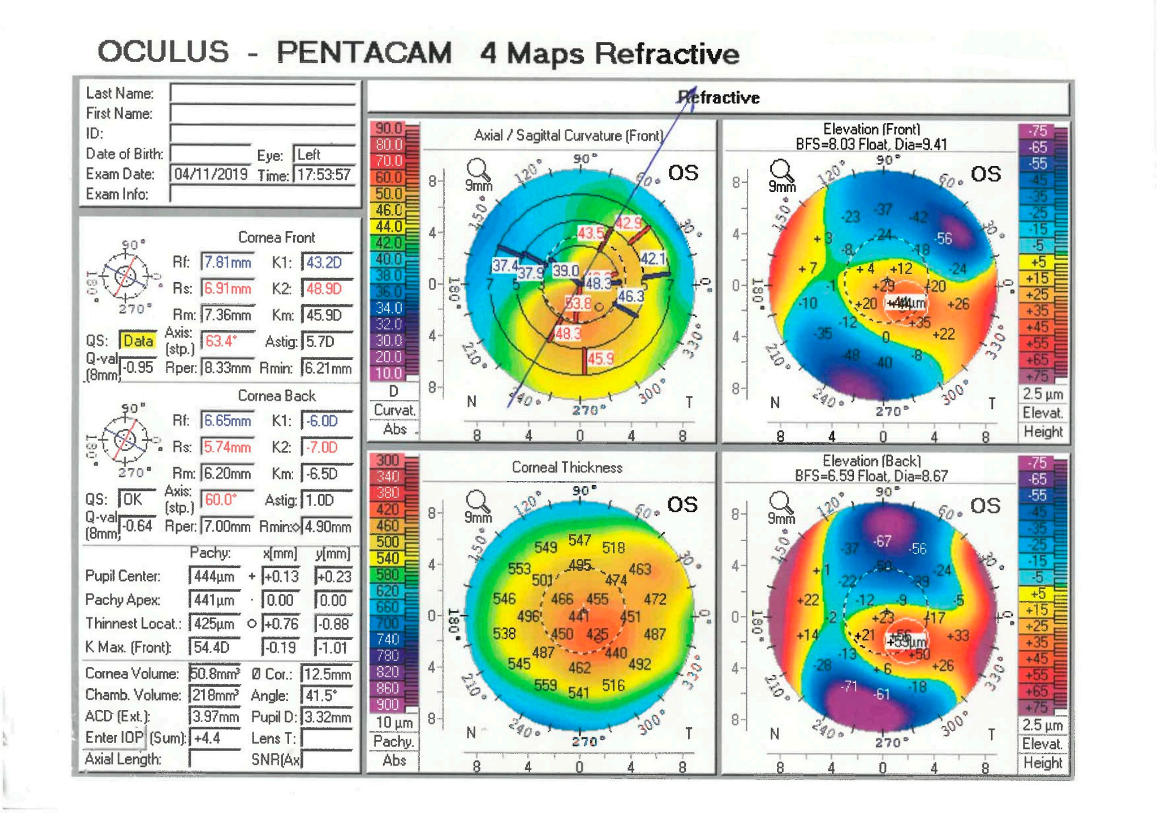

Supplementary Figure S6. Same patient, left eye: baseline Pentacam 4 Maps Refractive display obtained on 04 November 2019.

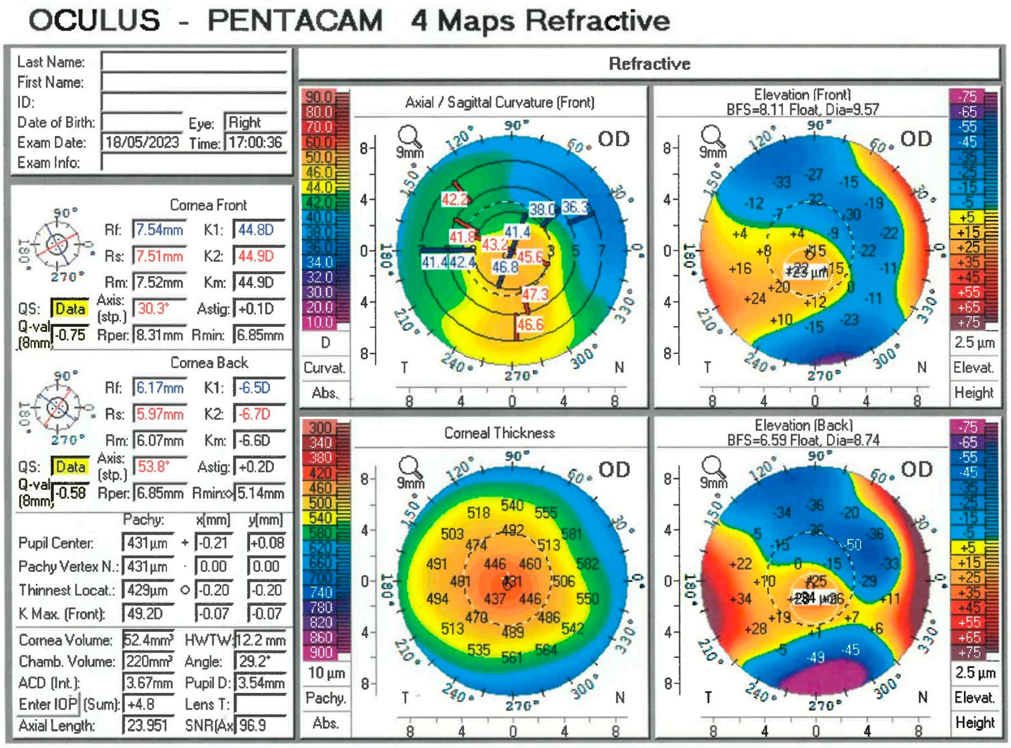

Supplementary Figure S7. Same patient, right eye: approximately 3-year postoperative Pentacam 4 Maps Refractive display obtained on 18 May 2023, showing marked reduction of anterior corneal astigmatism and a more regular central curvature pattern.

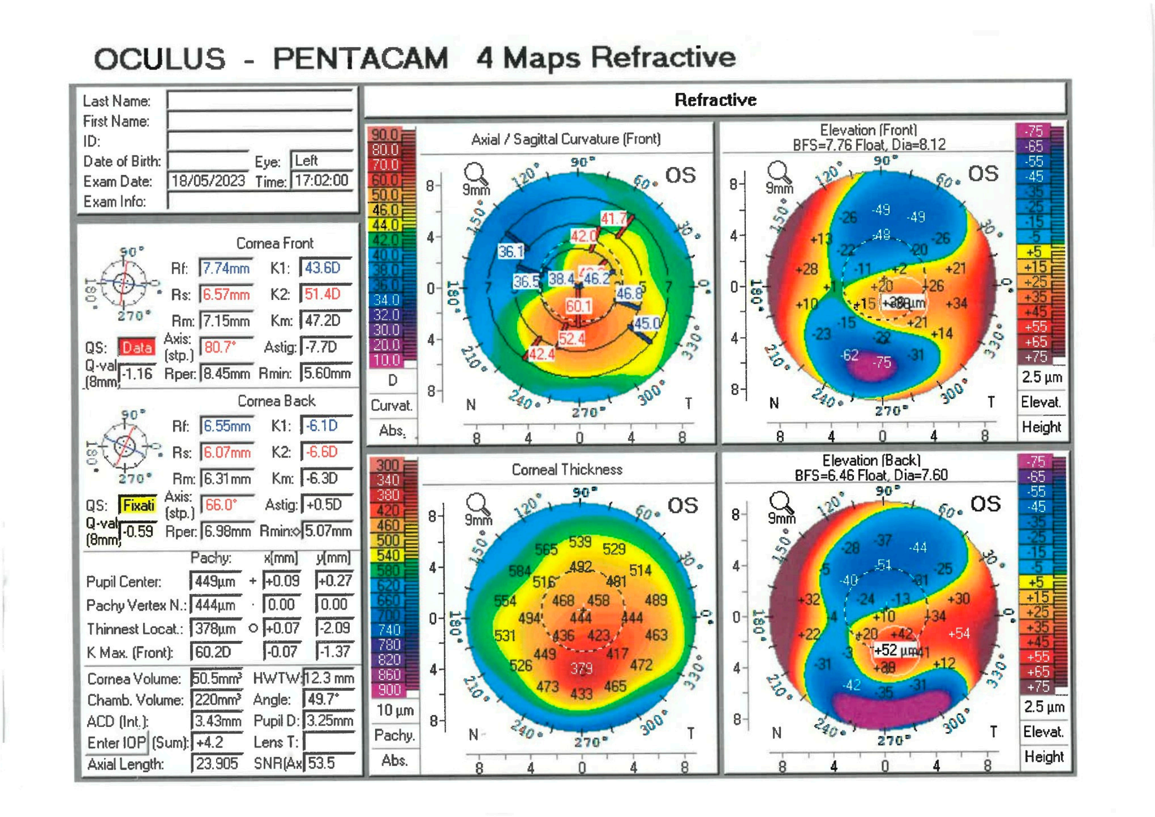

Supplementary Figure S8. Same patient, left eye: approximately 3-year postoperative Pentacam 4 Maps Refractive display obtained on 18 May 2023, illustrating residual inter-eye variability despite redistribution of the corneal curvature pattern.

Note: these images are illustrative and do not replace cohort-level statistics in the main manuscript.
